# Our Preliminary Experience with LMA C-Trach

**Published:** 2009-06

**Authors:** V N Swadia, Mamta G Patel

**Affiliations:** 1 Professor & Head, Dept. of Anesthesiology, Medical college, Baroda; 2 Assistant Professor, Dept. of Anesthesiology, Medical college, Baroda

**Keywords:** Equipment, airway; LMA CTrach and ETT, Anaesthesia:GA

## Abstract

**Summary:**

The LMA CTrach is a variant of the intubating LMA. It provides visualization of larynx during intubation and is a promising addition to airway management cart.

A preliminary study of 20 patients posted for elective surgery requiring GA were enrolled for the study. Their age ranged from 16-60 years, weight ranged from 45 to 65 kg, and they were belonging to ASA PS I & II with normal airways. Conventional general anaesthesia was administered in all the cases. The success rate and attempt of insertion of CTrach and ETT were observed. Viewing of larynx was graded as good, acceptable and poor. Requirement of manipulations was also noted down. Time for insertion of CTrach and ETT, view of larynx and complete procedure were noted down.

We successfully inserted LMA CTrach at first attempt in all the patients within 36.75 ± 2.12 sec and ventilation was possible in all cases. We were able to view larynx in majority of cases (95%), while in 1 patient (5%), we could not view the larynx even after manipulations, although ET intubation was successful in that case. Time required for viewing of larynx was 240.2 ± 10.5 sec. Manipulation of LMA was required in 40% cases to obtain good view. ET intubation was done at first attempt in all the patients within 60.5 ± 5.15 sec. The time required for complete procedure was 347.75 ± 10.55 seconds. None of our patient had any complications and haemodynamic parameters and SpO_2_ remained within normal limits throughout the procedure. The post operative period was uneventful. We successfully ventilated and intubated all the patients using LMA CTrach.

## Introduction

LMA C Trach, a modified version of Intubating LMA (ILMA), allows continuous video-endoscopy of the tracheal intubation procedure through LMA.^1^–^3^ It is a new system for airway management and endotracheal intubation (ETI). This system comprises of LMA with fibreoptic channels and detachable LCD viewer. So, this system enables simultaneous viewing of larynx and process of ET intubation via LMA.

Since its introduction in April 2005 and modification in December 2005, this device seems to be a promising addition to airway management cart. This LMA C Trach system also enables higher success rate of ventilation and intubation in difficult airway situations.^2^

Here, we undertook this preliminary study to evaluate success rate of Insertion of LMA C Trach, Viewing of larynx and Endotracheal intubation.

## Methods

After obtaining institutional approval and written informed consent, total 20 adult patients posted for elective procedures requiring GA were enrolled for the study. This is our preliminary study, so we have taken only 20 cases.

Their age ranged from 16-60 years, weight ranged from 30-65 kg and they had ASA PS I and II. Patients who were pregnant, morbidly obese, non-fasted, who had gastro-esophageal reflux, delayed gastric emptying or severe respiratory disease, were excluded from the study. As this was our preliminary study, we did not include patients with anticipated difficult intubation cases (morbidly obese, patients with abnormal airway anatomy and upper esophageal pathology, Mouth opening less than 2 finger breadth) in this study.

## LMA C Trach system

**Fig 1:**
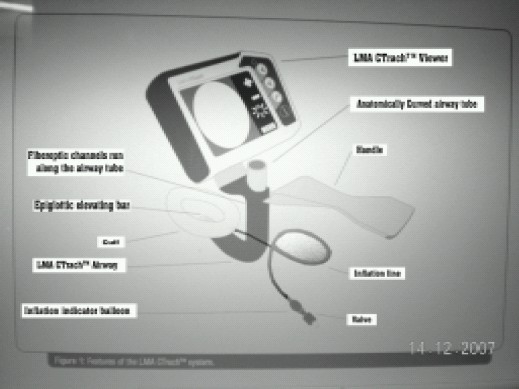
Fig 1 Photograph of LMA-CTrach

The LMA C Trach system is shown here in Fig 1. This system comprises of anatomically curved LMA with airway tube, LCD viewer, epiglottis elevating bar and endotracheal tube. The LMA C Trach has two in built fibreoptic channels to convey light and image. It has an epiglottic elevating bar and an aperture through which the anatomy, anterior to the bar can be viewed. The CTrach is designed so that mask aperture is located over glottis enabling view of laryngeal structure. The shape of this device is based on MR imaging of human adult airway. The magnetic latch connector at the top of LMA C Trach connects LCD viewer. The viewer has a high resolution 86 mm LCD color display screen and a thumbwheel for adjusting the focus. It also has a rechargeable battery for up to 30 min of continuous use with a charger cradle for recharging the viewer.

## Pre insertion preparation

Selection of size of LMA C Trach and ETT was done as per patient's body weight as follows-

Size 3 LMA C Trach & 6.0 mm ETT-used for pts with BW-30-50 kg

Size 4 LMA C Trach & 7.0 mm ETT-used for pts with BW-50-60 kg

Size 5 LMA C Trach & 7.5 mm ETT- used for pts with BW->60 kg

Before insertion of LMA C Trach, the cuff of the LMA was fully deflated with the help of cuff deflator. Only posterior surface of LMA was well lubricated with the jelly to prevent obscuring of the fibreoptic channel port.

The LCD viewer was attached to LMA C Trach and focusing was adjusted by obtaining a sharp image of gauze piece or sheet of text held 1 cm in front of the Fibreoptic channel port. Then viewer was detached from the LMA.

## Anaesthesia and airway management:

We enrolled 20 adult patients, 16-60 years of age, ASA PS I & II, Mallampati's classification I & II, who required GA with oral ET intubation for elective surgery.

After confirming NBM status, all the patients were pre medicated with glycopyrrolate 0.01 mg.kg^−1^ IM 30 min before the induction of anaesthesia. Midazolam 0.03 mg.kg^−1^ and fentanyl 2mcg.kg^−1^ IV were given 2 min before induction.

After pre oxygenating the patients for 5 min, we induced general anaesthesia with propofol 2.5 mg.kg^−1^ IV and inhalation of sevoflurane (2% conc.) till loss of eyelash reflex. After confirming of face mask ventilation, vecuronium bromide 0.1 mg.kg^−1^ IV was given for muscle relaxation. Anaesthesia was maintained with sevoflurane +O_2_ +N_2_O during the procedure.

After adequate jaw relaxation, C Trach was inserted by authors who had more than 5 years of experience of insertion of various type of LMAs, the cuff of LMA was inflated with air to achieve airtight seal (size 3 = 20 ml/ size 4 = 30 ml and size 5 = 40ml) and Bain's circuit was connected and ventilation was confirmed by bilateral equal air entry.

**Fig 2:**
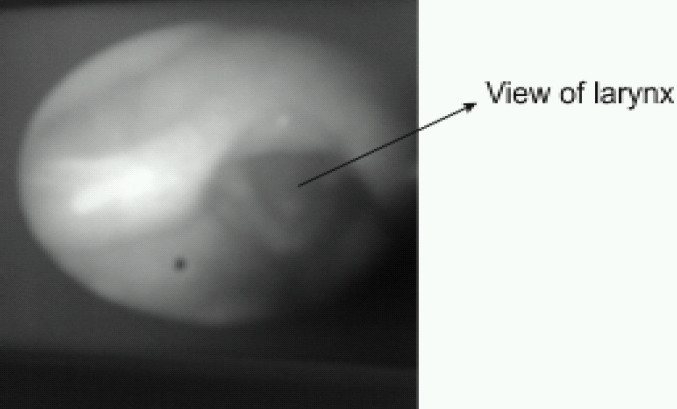
Viewing of larynx in LCD screen

Time and attempt required for insertion of C Trach were noted down. After satisfactory ventilation was achieved, the LCD viewer was connected to the C Trach and the view of the larynx was obtained as shown in Fig 2. During this period, ventilation via C Trach was continued.

The viewing of larynx was scored as Grade-1 (good view), Grade-2 (acceptable view) and Grade-3 (poor view). Ease of obtaining view on first attempt was scored as easy, difficult and impossible. In case of inability to view the larynx, manipulation of C Trach was done to obtain the view.

Success of viewing of larynx after manipulation was scored as

- View improved - Grade-1(good), Grade-2 (acceptable) and Grade-3(poor)
- No change
- Worsen/ Impossible

**Fig 3:**
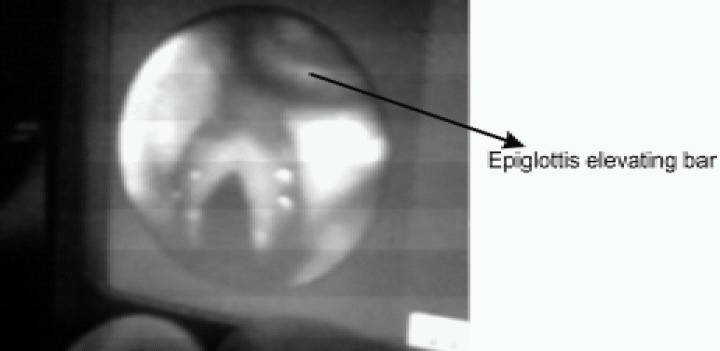
Elevation of Epiglottis elevating bar

**Fig 4:**
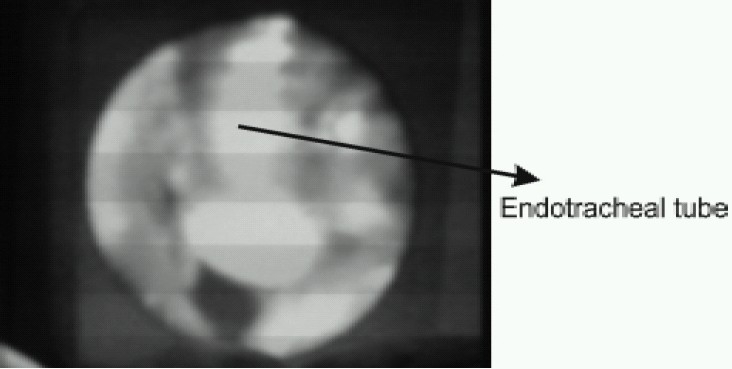
Insertion of ET tube

**Fig 5:**
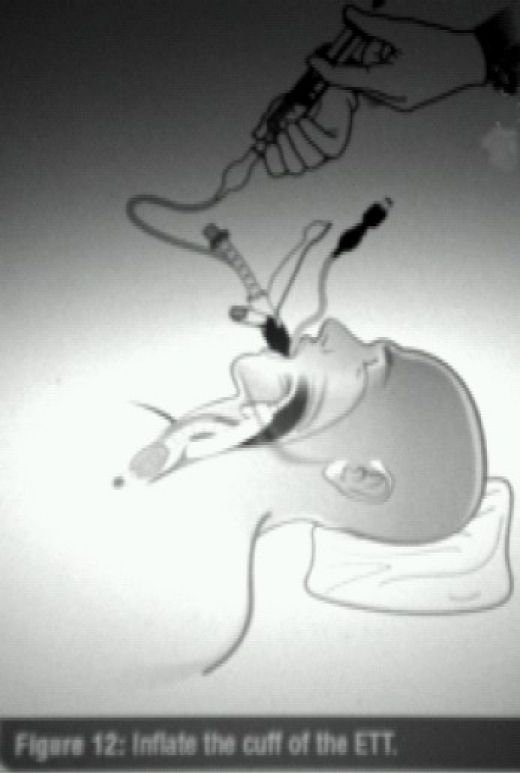
Complete process of LMA Trach

**Fig 6:**
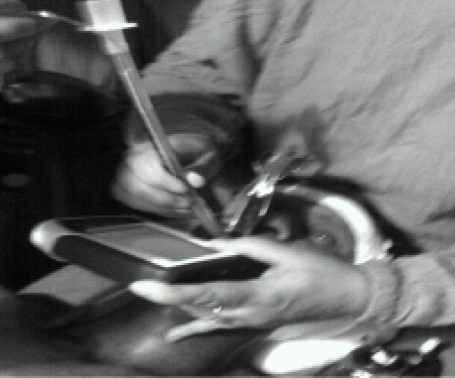
Process of Intubation via LMA-CTrach

After obtaining a best possible view (could see the vocal cords and laryngeal inlet in the centre of viewer), well lubricated ET tube was inserted through LMA C Trach. The blue colored epiglottis elevating bar (which elevates epiglottis at the time of tube insertion) was seen on the screen as seen in Fig 3. We used flexible, cuffed, wire reinforced silicone ET Tubes for all the patients. Correct placement of tube was confirmed by direct visualization on the LCD screen as in Fig 4 and by chest auscultation. We then inflated the cuff of ETT and detached the viewer, removed the ETT connector, deflated the CTrach cuff and removed the LMA CTrach over the ETT with the use of a stabilizer rod. The ETT connector was then replaced and connected to Bain's circuit for IPPV. Again, ventilation via ETT was confirmed by equal air entry. The Fig 5 and Fig 6 show complete process of intubation via LMA-CTrach.

### Various observations made in this study were as follow–

Time T1- Time required from picking up of LMA CTrach up to its Insertion and confirmation of placement of LMA.

Time T2- Time required from insertion of LMA up to achievement of view of larynx in the LCD Viewer.

Time T3- Time required from achievement of view of larynx up to insertion of ET tube through LMA.

Time T4- Total Time required from insertion of LMA and insertion of ETT through LMA.

We also observed conditions during the procedure like-

- No. of attempts for C Trach insertion & successful ventilation,
- Ease and quality of obtaining view of larynx,
- No. of attempts for intubation and
- No. of intubation without proper view through C Trach and
- Failed intubation (Max.3 attempts).

Intra operative monitoring was done for pulse rate, blood pressure, SpO_2_. Changes in vital parameters and SpO_2_, any complication occurred, were also noted. At the end of surgery, reversal was given using neostigmine 0.5mg.kg^−1^ and glycopyrrolate 10 mcg.kg^−1^ IV and extubation was done when the criteria was fulfilled.

Statistical analysis was done using Mean and SD to describe parametric data and Rate and Percentages for success rate.

## Results

Table-1 shows various demographic data and Table-2 shows types of surgery in study. The time required for insertion of LMA CTrach system is shown in Table-3.

**Table 1:** Demographic Data

Age (years)		28 ± 2.55
Sex(M:F)		1:1
Weight (kg)		50.5 ± 5.45
ASA PS	I	85%
	II	15%
Mallampati grade	I	90%
	II	10%
Duration of surgery		2.5 ± 0.35 hours

Values are mean ± SDor percentage

**Table 2:** Types of surgery, n(%)

Surgery	No of cases (%)
Mastoidectomy	10 (50%)
Septoplasty	07 (35%)
Tympanoplasty	01 (5%)
Excision of submandibular gland	01 (5%)
Excision of nasal polyp	01 (5%)

**Table 3:** Time required for insertion of LMA CTrach system

Procedure Time	Our study	Liu EHC et al
T1- Time for insertion of CTrach (seconds)	36.75 ± 2.12	26(20-33)
T2- time to achieve view of larynx (seconds)	240.20 ± 10.05	65 (30-141)
T3- Time for successful intubation (seconds)	60.5 ± 5.15	55 (48-65)
T4- time for complete procedure (seconds)	347.75 ± 10.55	166 (144-233)

We successfully inserted CTrach LMA at first attempt in all the patients in 36.75 ± 2.12 sec. We were able to view larynx in majority of cases (19 patients), while in 1 patient, we could not view the larynx, although ET intubation was successful in that case. Thus, insertion of ETT was at first attempt in all the patients in our study. The conditions observed during the procedure are as shown in Table-4. Vital parameters and oxygen saturation in our study are as shown in the Table-5. Fall in systolic BP was observed after 10 min in 30% cases which was easily managed with O_2_, IV fluids and by decreasing concentration of sevoflurane. None of our patients developed cyanosis, laryngospasm or local injury related to procedure. Only in one patient, coughing was observed during intubation. None of our patients developed sore throat or other complication in the postoperative period.

**Table 4:** Conditions of insertion of CTrach & ETT

Variables		No & % of pt
**A.Insertion of LMA CTrach**		
1. No. of attempt	First attempt	20 (100%)
2. Ease of ventilation via LMA	Easy	20 (100%)
**B. Viewing of larynx**		
1. Quality of view —	Good	12 (60%)
	Acceptable	07 (35%)
	Poor	01 (5%)
2.Manipulation of LMA required		08 (40%)
3.Success of view after manipulation	Good	5 (62.5%)
	Acceptable	2 (25%)
	Poor	1 (12.5)
**C.Insertion of ETT**		
1. Attempt	First attempt	20 (100%)
2.No. of TI without proper view	-	01 (5%)
3.Failed intubation through CTrach	-	00

**Table 5:** Changes in hemodynamic parameters and O_2_ saturation (Mean ± SD)

Parameter	Pulse rate Per min	SBP mm of Hg	DBP mm of Hg	SpO_2_ (%)
Base line	91.45 ± 15.74	120.1 ± 13.82	76.8 ± 11.41	98.4 ±0.50
1 min	90.5 ± 18.10	114.25 ± 12.85	77.35 ± 10.67	98.7 ± 0.73
5 min	94.6 ± 18.50	110.6 ± 13.80	76.5 ± 10.53	98.65 ± 0.81
10 min	90.5 ± 13.75	101.5 ± 12.19	68.3 ± 11.47	98.35 ± 0.49
15 min	86.1 ± 17.77	99.05 ± 10.15	66.3 ± 10.46	98.4 ± 0.82
20 min	85 ± 16.11	103.9 ± 11.63	70.6 ± 11.49	98.3 ± 0.98

The anaesthesia was uneventful in all the patients.

## Discussion

The intubating LMA (ILMA) was invented and bioengineered by Dr. Archie Brain in 1997 using MR scans of the adult airway.^4^ This device required at least three hands to intubate trachea and thus became cumbersome procedure. Then, the LMA CTrach was developed to minimize the technical effort required by the user and fiberoptic components are integrated into the ILMA for direct view of the larynx. Thus, LMA CTrach is a new system for airway management and ET intubation.

The LMA CTrach is indicated as an alternative for achieving and maintaining an airway. It seems to be a promising addition to airway management cart and can be used in both anticipated and unexpected difficult airway situations. The use of this system is not suitable in case of esophageal or pharyngeal pathology and prone position.

In our study, we successfully inserted CTrach in all the patients at first attempt in 36.75 ± 2.12 sec. In our study, the trained anaesthesiologist experienced in insertion of CLMA & PLMA only introduced C Trach LMA. Our results are in consonance with that of Liu EHC et al^1^ and that of Timmermann et al^2^.

Considering Viewing of larynx, we obtained good view (grade-1) in 60% cases and acceptable view in 35% patients (grade-2). However, manipulation of CTrach LMA was required in 8 (40%) patients to obtain good view in our study and View was improved after manipulation. Only in one patient (5%), view was poor (grade-3). Neither manipulation nor focusing could improve the view and we decided to intubate without vision and were successful in doing so at first attempt via CTrach without proper view.

Liu et al^6^ had observed that, out of 100 patients, in 84% patients good view was achieved and 16% cases required manipulations of LMA. Timmermann^2^ et al observed that, manipulations required in 63.3% cases. According to them the most frequent causes of poor image quality in 28.3% cases were secretions in front of the lens and an inadequate light intensity. Dhonneur et al^7^ had observed that, 49% of patients required manipulation of CTrach resulting in increased duration of tracheal intubation by 57 seconds as compared to direct laryngoscopy (DL) in their study. But oxygenation was of better quality in patients managed with CTrach than DL. They observed that, LMA CTrach is only airway that allows ventilation during in-tubation attempts and allows for less stressful situations. In our study, time to achieve view of larynx was more (240.2 ± 10.5 sec) in comparison to Liu^6^ et al. We attribute this to our learning phase. In our study, we intubated all the patients successfully at first attempt. Even in case of poor view, we intubated the patient without vision at first attempt. We did not observe any failed intubation.

Liu et^1^ al had observed that, out of 100 patients, in 14 patients intubation was done without proper view at first attempt and in 2 patients after three attempts. They were unable to centralize the view of larynx and to align the CTrach with the larynx despite several manipulations. In their study, conventional intubation with Macintosh laryngoscope was carried out in 2 patients (unable to direct ETT into the trachea after three attempts). Timmermann et al^2^ also observed that tracheal intubation (TI) was successful at first attempt in 71.7% cases. TI without proper view was done in 26.6% cases. In one patient, they failed for intubation and had to use conventional laryngoscopy.

In our study, intubation was carried out within 60.5 ± 5.15 sec and the time is comparable with other studies. Total time required for complete procedure is more in our study (347.75 ± 10.55 sec) as compared to Liu et al as per Table-3. This might be because of more time was taken in viewing of larynx.

Hemodynamic parameters and SpO_2_ were remained within normal limits in our study. None of our patients had any complication related to procedure. Only in one patient, coughing was observed at the time of intubation, which might be because of inadequate effect of muscle relaxant.

Thus, we successfully ventilated and intubated all the patients using LMA CTrach. The CTrach enables nearly continuous ventilation and oxygenation of the patient during the intubation process.

In this preliminary study, we could successfully use the CTrach system for intubation and now we can proceed to use this system for cases with difficult airway.
